# The impact of a lack of mathematical education on brain development and future attainment

**DOI:** 10.1073/pnas.2013155118

**Published:** 2021-06-07

**Authors:** George Zacharopoulos, Francesco Sella, Roi Cohen Kadosh

**Affiliations:** ^a^Wellcome Centre for Integrative Neuroimaging, Department of Experimental Psychology, University of Oxford, Oxford OX2 6GG, United Kingdom;; ^b^Centre for Mathematical Cognition, Loughborough University, Loughborough LE11 3TU, United Kingdom

**Keywords:** mathematical education, GABA, plasticity, middle frontal gyrus

## Abstract

Formal education has a long-term impact on an individual’s life. However, our knowledge of the effect of a specific lack of education, such as in mathematics, is currently poor but is highly relevant given the extant differences between countries in their educational curricula and the differences in opportunities to access education. Here we examined whether neurotransmitter concentrations in the adolescent brain could classify whether a student is lacking mathematical education. Decreased γ-aminobutyric acid (GABA) concentration within the middle frontal gyrus (MFG) successfully classified whether an adolescent studies math and was negatively associated with frontoparietal connectivity. In a second experiment, we uncovered that our findings were not due to preexisting differences before a mathematical education ceased. Furthermore, we showed that MFG GABA not only classifies whether an adolescent is studying math or not, but it also predicts the changes in mathematical reasoning ∼19 mo later. The present results extend previous work in animals that has emphasized the role of GABA neurotransmission in synaptic and network plasticity and highlight the effect of a specific lack of education on MFG GABA concentration and learning-dependent plasticity. Our findings reveal the reciprocal effect between brain development and education and demonstrate the negative consequences of a specific lack of education during adolescence on brain plasticity and cognitive functions.

## Significance

Our knowledge of the effect of a specific lack of education on the brain and cognitive development is currently poor but is highly relevant given differences between countries in their educational curricula and the differences in opportunities to access education. We show that within the same society, adolescent students who specifically lack mathematical education exhibited reduced brain inhibition levels in a key brain area involved in reasoning and cognitive learning. Importantly, these brain inhibition levels predicted mathematical attainment ∼19 mo later, suggesting they play a role in neuroplasticity. Our study provides biological understanding of the impact of the lack of mathematical education on the developing brain and the mutual play between biology and education.

Educational decisions have a long-lasting impact on both the individual and wider society (1). Mathematical education and attainment has been associated with several quality-of-life indices, including educational progress, socioeconomic status, employment, mental and physical health, and financial stability (2–5). In several countries, such as the United Kingdom and India, 16-y-old adolescents as part of their advanced (i.e., A-level) subjects can choose to stop studying math. The consequences of not choosing math as an A-level subject can be significant. When controlling for potential confounding factors such as socioeconomic status it emerged that the decision to not study math as an A-level subject can lead to an 11% decrease in financial income compared to those who choose to study math as part of their A-level curriculum. No other A-level subject category is associated with such a wage premium (6). In addition, previous studies highlighted the cognitive, emotional, and societal factors that are associated with mathematical education (7, 8).

In recent years, there has been significant interest in the investigation of the neural substrates of mathematical cognition and education, and frontal and parietal regions have been repeatedly highlighted as key regions (9–13). Despite the advancement of our knowledge on the neurobiological underpinnings of math abilities, little is known about whether and how they are involved in a lack of mathematical education. At the neurobiological level, the lack of math education could impact neural changes in regions that are involved in skill acquisition of math, primarily in frontoparietal regions (“plasticity account”). This process can be subserved by neurotransmitter concentrations that preceded anatomic changes (14). However, such differences may exist before the continuation of math education and represent baseline differences at the time of the educational decision not to study vs. to study further math (“biomarker account”).

**Fig. 1.**
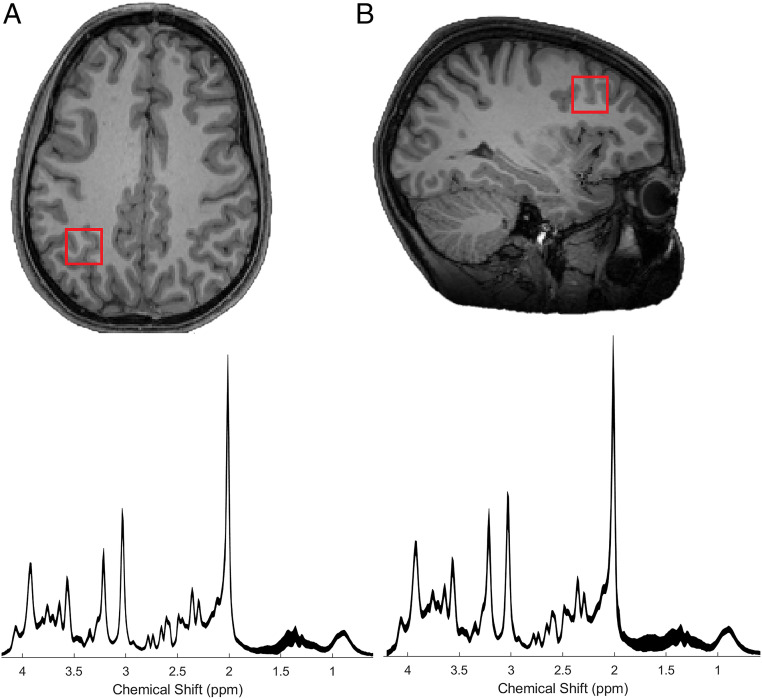
Positions of the volumes of interest displayed in a representative T1-weighted image for the (*A*) IPS and (*B*) MFG, on axial and sagittal slices, respectively. Average spectra from each of these regions are shown below (thickness corresponds to ±1 SD from the mean) (chemical shift expressed in parts per million, ppm, on the *x* axis).

Using single H-magnetic resonance spectroscopy (MRS), we scanned two previously defined key regions involved in numeracy: the intraparietal sulcus (IPS) and the middle frontal gyrus (MFG) (Fig. 1). We also examined their functional connectivity using resting-state functional MRI (for reviews see refs. 15–19). Such an approach allowed us to examine the role of γ-aminobutyric acid (GABA) and glutamate, the brain major inhibitory and excitatory neurotransmitters, respectively. Brain inhibition and excitation levels are thought to be critical in triggering the onset and defining the duration of sensitive periods of a given function, during which the neural system is particularly plastic in its response to environmental stimulation (20). It is thought that this is achieved by a shift in the ratio of intrinsic and spontaneous activity and activity in response to the environmental stimulation, whereby the intrinsic and spontaneous activity is reduced and the activity in response to the environmental stimulation is increased (21). Although very early in development, GABA functions as an excitatory neurotransmitter (22), during adolescence GABA and glutamate function as the main inhibitory and excitatory neurotransmitters, respectively, and previous studies have shed some light on the actions of these two neurotransmitters during adolescence. For example, compared to early childhood where there is a peak synaptic density, but the synaptic density is significantly reduced during adolescence (even more so compared to adulthood) and such synaptic pruning is thought to be underpinned by glutamatergic-mediated synaptic mechanisms of long-term potentiation and depression (23). Moreover, previous studies have shown that GABA matures during adolescence, and frontal lobe GABA receptors reach adult levels late in adolescence with lower GABA levels being associated with poor cognitive functioning during adolescence (24, 25).

In the present study, rather than examining a general lack of academic education, which could stem from several confounding factors (e.g., socioeconomic status, lack of learning materials, insufficient educational infrastructure, cultural differences), we specifically examined the lack of math education. As mentioned earlier, in the United Kingdom, 16-y-old adolescents can choose to cease their mathematical education while still being enrolled in other nonmathematical academic education. This allowed us to better control for these confounds by recruiting participants from similar educational systems who differ specifically in their math education.

Based on the existing literature reviewed previously, we hypothesized that the lack of mathematical education would be associated with reduced GABA and/or increased glutamate. While both left and right frontoparietal regions were shown to underpin numerical processing (13, 26, 27) in the present study, we focused on the left frontoparietal regions due to their central role in mathematical learning (28–31). Our decision to a priori select the left IPS and MFG was based on the following reasons: First, the left IPS and MFG are frequently reported in neuroimaging studies that examined arithmetic, including a metaanalysis (10). Second, previous studies in the field of numerical cognition have shown the involvement of those brain regions in cognitive training (32–34). Third, brain stimulation studies have suggested a causal role of the MFG in algorithmic learning and the IPS in learning concerning more low-level computation (numerosity, symbolic representation) (30, 35, 36). Using classification approaches, we discerned the differences in these neurotransmitters in adolescents who lack further math education (A-level nonmathematics) vs. those who underwent further math education (A-level mathematics). To dissociate the plasticity account from the biomarker account, we examined in a second experiment an independent cohort of students who made the same decision but who had not yet started their A level. Such a design allowed us to understand the exact role of frontoparietal GABA and glutamate, the main determinants of neuroplasticity and cognitive functions, during this critical developmental and educational stage.

## Results

### Contrasting Nonmath vs. Math Students.

**Fig. 2.**
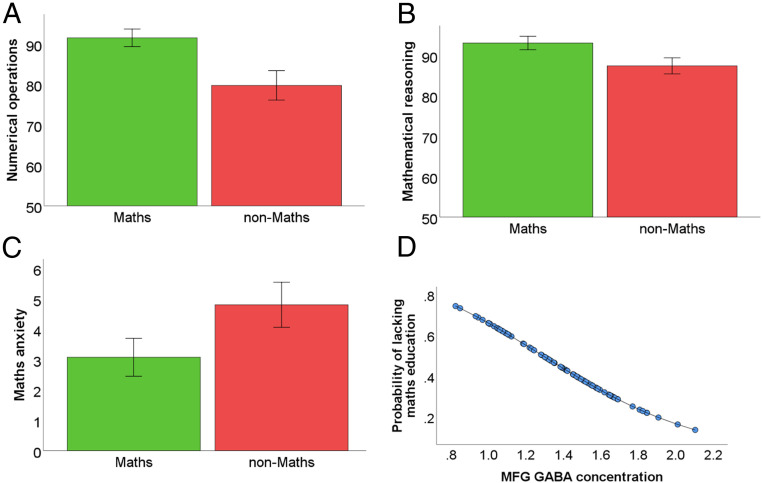
Behavioral performance and GABA-based classification of whether a student is currently lacking math education. Behavioral results showing worse performance for adolescents who did not study math compared to those who did study math on (*A*) numerical operation attainment test and (*B*) mathematical reasoning attainment test. (*C*) Those who did not study math scored higher on a test that assessed math anxiety. Error bars represent 95% confidence intervals. (*D*) MFG GABA classified math education; predicted probabilities of those currently lacking math education (*y* axis) plotted against MFG GABA concentrations (*x* axis).

In line with previous studies (7, 8, 37), we identified that those who ceased to study A-level mathematics, compared to those who continued to study A-level mathematics, showed lower performance in tests involving numerical operations [Fig. 2*A*, *t* (84) = −5.27, *P* < 0.001, Cohen’s d = −1.18] and mathematical reasoning [Fig. 2*B*, *t* (83) = −4.61, *P* < 0.001, Cohen’s d = −0.99], but scored higher on a test that assessed math anxiety [Fig. 2*C*, *t* (84) = 3.3, *P* = 0.001, Cohen’s d = 0.71]. For the descriptive statistics of these cognitive and emotional differences, see *SI Appendix* 1.

We then used GABA and glutamate concentrations in the MFG and IPS to classify students based on their present lack of math education (nonmath students vs. A-level math) using a binary logistic regression. Lower MFG GABA concentrations increased the likelihood that a student lacked math education rather than continued their math education [Fig. 2*D*, *n* = 83, standardized beta(β) = −0.3, *P* = 0.009, Exp(pβ) = 0.524, Exp(β) = 0.742]. This finding survived multiple comparisons [Benjamini–Hochberg adjusted *P* = 0.037, based on the two regions of interest (IPS/MFG) × two neurochemicals (GABA/glutamate)].

To examine whether GABA’s role is regionally and neurochemically specific in classifying the lack of a present math education, we ran a binary logistic regression with the predictors of MFG GABA [*n* = 82, β = −0.31, *P* = 0.007, Exp(pβ) = 0.49, Exp(β) = 0.74], MFG glutamate [*n* = 82, β = −0.01, *P* = 0.9, Exp(pβ) = 0.97, Exp(β) = 0.99], and IPS GABA [*n* = 82, β = 0.14, *P* = 0.19, Exp(pβ) = 1.39, Exp(β) = 1.15]. As the results indicate, only the MFG GABA predictor was significant. These results allowed us to conclude that our finding is regionally and neurochemically specific.

To examine whether the contribution of MFG GABA is confounded by math ability or math anxiety, we ran a binary logistic regression classifying whether a student lacks math education based on numerical operations [*n* = 80, β = −0.3, *P* = 0.023, Exp(pβ) = 0.38, Exp(β) = 0.74], mathematical reasoning [*n* = 80, β = −0.24, *P* = 0.074, Exp(pβ) = 0.47 Exp(β) = 0.79], and math anxiety [*n* = 80, β = 0.12, *P* = 0.26, Exp(pβ) = 1.46, Exp(β) = 1.13], and MFG GABA [*n* = 80, β = −0.23, *P* = 0.023, Exp(pβ) = 0.48, Exp(β) = 0.79]. These findings support the notion that math ability measures, but not math anxiety, successfully classify whether an adolescent lacks a math education program. More importantly, the findings highlight the contribution of MFG GABA in classifying whether an adolescent does not study math above and beyond the other aforementioned common determinants. Further analyses examined the effect of potential confounds. First, we excluded the role of gender in the observed results (*SI Appendix* 2). Second, we revealed that students who lack vs. those who did not lack math did not differ in their A-level education duration, age at scanning, and matrix reasoning (*SI Appendix* 3), and that MFG GABA successfully classified those students who lack math education even after controlling for the total number of enrolled A-level subjects (*SI Appendix* 3). Third, MFG GABA successfully classified students who lack math education at this age, even after controlling for the choice of biology, chemistry, and physics, while it failed to classify students who lack a physics, biology, or chemistry education at the same developmental stage.

Furthermore, we employed resting fMRI to investigate whether the lack of math education is associated with the functional connectivity between the left MFG, the region in which the main neurochemical finding was obtained, and the rest of the brain, using a seed-to-voxel method (*Materials and Methods*). However, no significant clusters were obtained, even after lowering the threshold to that of a liberal one (voxel-wise *P* value = 0.01).

**Fig. 3.**
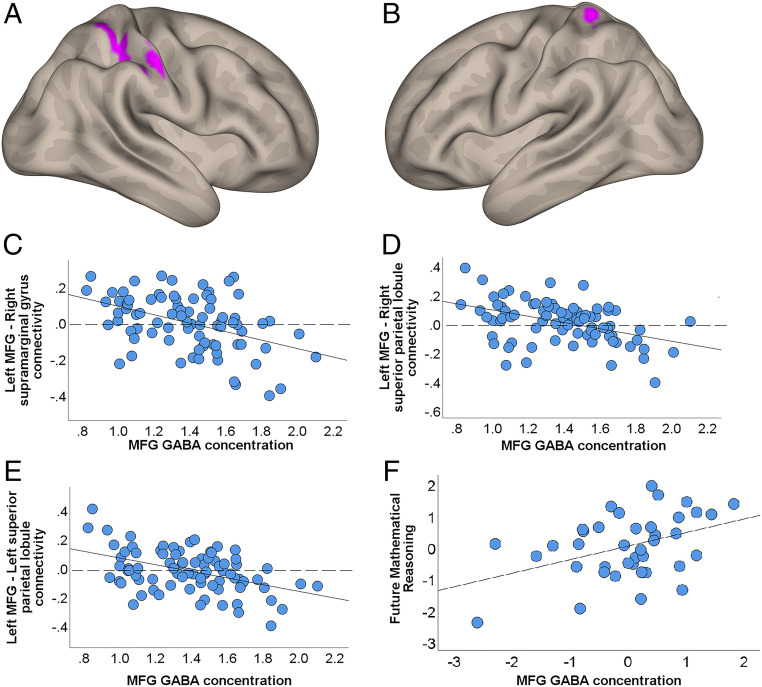
MFG GABA is associated with frontoparietal functional connectivity and predicts future math performance. Regions negatively connected to left MFG as a function of MFG GABA concentration included (*A*) the right supramarginal gyrus and the right superior parietal lobule and (*B*) the left superior parietal lobule. Scatterplots depict the negative associations between MFG GABA concentration and MFG-based brain connectivity in three parietal clusters: (*C*) left MFG–right supramarginal gyrus, (*D*) left MFG–right superior parietal lobule, and (*E*) left MFG–left superior parietal lobule. Dotted vertical lines in *C*–*E* correspond to the zero-connectivity point. (*F*) MFG GABA concentration predicted future mathematical reasoning after controlling for mathematical reasoning scores when MFG GABA concentration was acquired ∼19 mo before. We plotted the residuals on the *y* axis and *x* axis after controlling the variance of the other predictors in the regression model described in the text.

We then employed resting fMRI to investigate whether MFG GABA concentration regulates functional connectivity between the left MFG and the rest of the brain (*Materials and Methods*). The identified associations with the MFG GABA and the functional connectivity with this region were restricted to the parietal cortex and included three clusters: the right supramarginal gyrus (Fig. 3*A*, x = 56, y = −22, z = 48, k = 300 voxels, positive false discovery rate [pFDR] = 0.016) and the bilateral superior parietal lobules (Fig. 3*A*, x = 30, y = −46, z = 60, k = 231 voxels, pFDR = 0.024; Fig. 3*B*, x = −26, y = −42, z = 68, k = 199 voxels, pFDR = 0.028). In all of these cases, higher MFG GABA concentrations were associated with negative connectivity, while low MFG GABA concentrations were associated with positive connectivity (Fig. 3 *C*–*E*).

### Dissociating the Plasticity and Biomarker Accounts.

At this stage, the relation between MFG GABA concentrations and math education might reflect baseline differences before studying A-level math. Such baseline differences, for example, might reflect differences in cortical maturation (20), which could prove to be advantageous when studying math, and thus impacting the likelihood of continuing their math education. To investigate this possibility, we examined a new cohort who had already decided whether they would stop studying math as part of their A-level curriculum but were at that time engaged in math education (pre-A level).

Pre-A-level students who had chosen to stop studying math compared to those who had decided to continue studying math show lower performance on tests that included numerical operations [*t* (40) = −5.03, *P* < 0.001, Cohen’s d = −1.67] and mathematical reasoning [*t* (40) = −4.1, *P* = 0.001, Cohen’s d = −1.37]. However, the difference between both groups in terms of math anxiety was not significant [*t* (40) = 0.91, *P* = 0.4, Cohen’s d = 0.28]. For the descriptive statistics of these cognitive and emotional differences, see *SI Appendix* 1. The biomarker account did not receive support as the classification of the nonmath vs. math educational decision before starting to study math based on MFG GABA was not significant [*n* = 36, β = 0.14, *P* = 0.42, Exp(pβ) = 1.33, Exp(β) = 1.14]; for further analysis to exclude potential artifacts see *SI Appendix* 4.

### MFG GABA Predicts Future Mathematical Ability.

The plasticity account posits that the decreased MFG GABA concentration may reflect a lack of an advanced math education, which would otherwise benefit better math acquisition. Taking the results of the two preceding sections together, a corollary from the plasticity account is that the MFG GABA concentration will predict future math attainment. To test this hypothesis, we asked the students to complete the standardized numerical operations and mathematical reasoning tests 19.37 mo (SD = 3.94) after we acquired their MFG GABA concentration. Using regression analysis, we assessed whether MFG GABA in the A-level students could predict future math attainment while controlling for math attainment when the MFG GABA was acquired. We also controlled for other confounding factors (age during neuroimaging and behavioral data acquisition [T1], age during the second testing [T2], and group [lacking vs. not lacking math education]). The numerical operations score at T2 was predicted by numerical operations at T1 [β = 0.51, *t* (33) = 4.1, *P* < 0.001] and by math group [β = −0.43, *t* (33) = −3.63, *P* = 0.0005, a directional hypothesis based on our results at T1], but not by MFG GABA concentration [β = 0.06, *t* (33) = 0.59, *P* = 0.56]. The mathematical reasoning score at T2 was predicted by mathematical reasoning at T1 [β = 0.61, *t* (33) = 5.85, *P* < 0.001], and there was a trend with the factor group [β = −0.16, *t* (33) = −1.52, *P* = 0.068, a directional hypothesis based on our results from T1]. Most notably, the MFG GABA concentration at T1 was a significant predictor of the mathematical reasoning 19 mo later [Fig. 3*F*, β = 0.25, *t* (33) = 2.59, *P* = 0.014, Benjamini–Hochberg adjusted *P* = 0.028, based on two comparisons, i.e., numerical operations and mathematical reasoning]. See *SI Appendix* 5 for further analysis.

## Discussion

In the present study, we examined the impact of the lack of math education on brain development and future attainment. Using MRS, we scanned the left MFG and IPS, two previously defined key regions involved in numeracy, and observed three main findings: 1) adolescents who lack math education exhibited a decrease in GABA levels within the MFG compared to those who receive math education; 2) these differences were not present when deciding to cease math education, but before this action took place; and 3) MFG GABA levels predicted future mathematical reasoning when our sample was reassessed ∼19 mo apart.

We found that MFG GABA concentration could classify whether A-level students are presently lacking math education. Low MFG GABA concentrations increase the likelihood of not undergoing math education as a result of not choosing math as one of one’s A-level subjects. Notably, this finding was specific to math education and was not explained by other A-level subjects that are usually taken by those who are enrolled in math education such as biology, chemistry, and physics, or by the number of A-level subjects studied by the individuals (*SI Appendix* 3). GABA and glutamate within the IPS and glutamate within the MFG were not successful classifiers, and in further analyses, we showed that our results are specific to GABA and the MFG. Moreover, while we demonstrated that relevant cognitive and emotional factors, namely, math ability and math anxiety, differ between A-level students who lack vs. A-level students who do not lack math education, similar to previous studies (7, 8, 37), MFG GABA could still successfully classify whether A-level students lack math education even when we took into account the variance explained by these behavioral measures.

The neurochemical concentration within the MFG, but not within the IPS (a key region in numerical cognition), successfully classifies whether A-level students lack math education. The parietal cortex plays a crucial role in low-level computation, symbolic and nonsymbolic representation, and automatic execution after extensive practice (e.g., fact retrieval), whereas the prefrontal cortex is involved in more complex computations and algorithmic processing (36, 38–40), and it supports the allocation of mental resources during cognitive learning before automatic execution is reached (41). Such a distinction could explain our results, as A-level math students were involved in further mathematical education, which requires ongoing complex computation and algorithmic processing rather than low-level computation or automatization. While we acknowledge that the IPS is also found in some studies that examined the neural correlates of complex computation and algorithmic processing (26, 28, 42, 43), these findings do not provide causal evidence for the role of the IPS in the learning of these processes. A support for our suggestion is coming from brain stimulation studies over the MFG or IPS during a 5-d arithmetic learning, which found improved arithmetic learning and hemodynamic changes in the left MFG, but not the IPS (30). Furthermore, other brain stimulation studies have highlighted the role of the IPS in numerosity training and automatization, which requires low-level computation (35, 36, 40, 44). However, our current explanation for the lack of IPS involvement in A-level mathematical learning is based on a null result, and could have originated as a result of other factors. For example, the involvement of different brain regions, such as the MFG and IPS, in mathematical learning might depend on the exact stage of development (29, 45–47).

By further examining whether MFG GABA concentration could classify the decision to choose advanced math education before being enrolled in the A-level program, we inferred that MFG GABA concentrations reflect a lack of further math education during adolescence, rather than the likelihood of the student choosing A-level math. Our conclusions are in line with previous studies that linked GABA to cognitive performance (48) and plasticity (49–51) and the role of the frontal cortex functions in high-level cognition (52–56), including cognitive learning (41). An additional support to our proposal that GABA concentration in the MFG reflects neural priming for better skill acquisition of math is our ability to predict future mathematical reasoning in a standardized mathematical attainment test using MFG GABA concentration values that we obtained ∼19 mo earlier. This result is supported by previous neuropsychological and functional MRI studies that have highlighted the role of the left MFG in reasoning and problem solving (57–59) (for the overlapped Montreal Neurological Institute [MNI] coordinates between the left MFG in our study and previous fMRI studies, see *SI Appendix* 5).

MFG GABA predicted future mathematical reasoning but not numerical operations. The numerical operations used in standardized tests, as in our testing battery, mainly emphasize the implementation of arithmetical procedure and the retrieval of arithmetic facts; the latter is mastered by students and is acquired in earlier years (60, 61) therefore leading to less room for future improvement. In contrast, mathematical reasoning emphasizes problem-solving abilities, which is a more malleable set of skills. Indeed, in our sample mathematical reasoning, but not numerical operations, significantly improved at T2 compared to T1 [mathematical reasoning: *t* (42) = 4.3, *P* < 0.001; numerical operations: *t* (42) = 0.52, *P* = 0.6]. Consequently, we tentatively suggest that the predictive capacity of MFG GABA becomes more relevant for skills that are more susceptible to change and plasticity rather than numerical operations, a more basic skill, which reached the level of proficiency in both groups.

What do these education-related GABA level differences reflect at the cellular level, and how may these differences affect plasticity? The learning of math is an evolving skill that is accumulated and refined over years, suggesting that its underlying sensitive period or plasticity extends beyond childhood and adolescence. Critically, however, sensitive periods for developing a skill are not an on/off process but are rather known to be plastic in terms of their onset, closure, duration, and intensity (20). Such plasticity mechanisms are thought to involve the maturation of parvalbumin cells, a positive subtype of GABA neurons (62), leading to an optimal excitation/inhibition ratio underlying plasticity and corresponding learning (20, 63–65). Notably, the parvalbumin cells account for 25% of GABAergic cells in the primate dorsolateral prefrontal cortex (66, 67). Therefore, a potential mechanistic explanation for our results is that the elevated GABA observed in the group that received math education vs. those who lack it reflect the maturation of parvalbumin cells. Moreover, individual variation in these prefrontal GABA levels predicted future mathematical reasoning, thus suggesting that the elevated GABA levels observed in the group that received an enhanced math education likely shifted the GABA prefrontal circuits from static to more plastic ones perhaps by increasing the amplitude or duration of plasticity (20). Subsequently, our findings propose testable hypotheses linking learning mechanisms from animal models described at the microcircuits profile to the lack of math education and corresponding math learning and underlie the macroscopic learning-dependent plasticity.

Work in animals has highlighted the role of GABA neurotransmission in neuronal coding and processing as it influences membrane potential, neuronal activity, and synaptic and network plasticity (49–51, 68, 69). Given this effect of GABA on neural networks, our study additionally examined whether individual variation in MFG GABA concentration is associated with the functional connectivity between the MFG and the rest of the brain. Our analyses revealed three parietal regions whose connectivity to the MFG was negatively associated with the amount of GABA concentration: the right supramarginal gyrus and the right and left superior parietal lobules. In all of these cases, high MFG GABA concentrations were associated with negative connectivity. These frontal and parietal regions have been repeatedly highlighted as structures involved in numerical cognition in both children and adults (9, 13), and we demonstrate here that such functional connectivities are linked to MFG GABA concentration during adolescence. Previous work discovered a negative association between regional GABA levels and resting-state brain connectivity (70, 71); for example, work in adults focused on the motor system found a negative association between GABA within the M1 and the connectivity strength of the motor network (72). Here, we found that higher frontal GABA concentration is related to negative frontoparietal connectivity. These results suggest that in adolescence, individuals with high MFG GABA levels exhibit increased negative associations between frontoparietal regions compared to individuals with low MFG GABA levels who exhibit increased positive associations. Since GABAergic interneurons are thought to coordinate the synchrony of neural circuits (71, 73, 74), a likely explanation at the neurobiological level is that regional GABA concentrations may determine the synchronicity valance of that region with other functionally connected regions, in this case, the frontoparietal network during adolescence. However, how GABA concentrations might impact the organization of neural networks during development remains unclear. One possibility is that the negative connectivity facilitates the allocation of more resources to the prefrontal regions due to their primary role in cognitive learning (41). Nonetheless, our data provide strong motivation to clarify the exact role of GABA in shaping the development of neural networks and to examine its potential causal effect, which could have implications for future brain-based interventions (75).

MRS-based neurochemicals like GABA or glutamate have been extensively examined in relation to their associations with laboratory-based tasks (47, 56, 63, 76–82). Moreover, MRS has been highlighted as a promising tool in the classification and prediction of brain-related diseases by focusing on neurochemical concentrations that preceded anatomic changes (14). Our findings extend the unique contribution of MRS from basic research and clinical applications to educational settings to gain insight into the effect of a specific lack of math education. From a societal perspective, this is highly relevant given differences between countries in their educational curricula. For example, in some countries, adolescents can stop their math education at the age of 16, while in other countries math is mandatory. We show that within a society such decisions can alter neural and cognitive development. This, in turn, can introduce an advantage to individuals and societies who introduced math education as mandatory until school graduation. In addition, one might further consider how the differences in opportunities to access education, as reflected especially during the Covid-19 pandemic, might impact neural and cognitive development.

In summary, our work highlights the role of a specific neurochemical, GABA, in education during adolescence, its association with brain connectivity, and its capacity to predict future math ability. These results raise further questions, such as the impact of MFG GABA differences on other cognitive abilities associated with math education, such as working memory and logic (43, 83, 84), whether mathematical education should be mandatory during adolescence, and whether ceasing to pursue mathematical education should be replaced with nonmath materials, such as reasoning and logic, to allow ongoing engagement of the MFG that has been implicated in such cognitive processes (59). A more integrative understanding of the role of neurochemicals in behavior and their potential impact on brain structures and neural activity could lead to better tools for improving education and understanding brain development and cognition.

## Materials and Methods

### Participants.

In experiment 1, we tested 87 A-level students {56 females, mean age in months = 202.7, SD = 4.7, range = [193 (16.1 y), 216 (18 y)]}. In the United Kingdom, A level is an advanced level subject-based qualification that can lead to university, further study, training, or work. In experiment 2, we tested 42 pre-A-level students {21 females, mean age in months = 172.31, SD = 3.71, range = [164 (13.7), 182 (14.92)]}. This group comprised prospective A-level students who indicated their decision of whether they would study math as part of their A-level curriculum. All participants completed two separate sessions; an imaging session that lasted ∼60 min and a mathematical assessment session that lasted ∼60 min. During the structural imaging and the MRS acquisition, participants merely watched the LEGO movie (85), and during the resting fMRI participants were asked to fixate on a white cross on a black background. All participants were predominantly right-handed, as measured by the Edinburgh Handedness Inventory (86). Exclusion criteria were any current or past neurological, psychiatric, or learning disability or condition that might affect cognitive or brain functioning; these were confirmed via self-report. As compensation, participants received a £35 Amazon or iTunes voucher.

The study was approved by the University of Oxford Medical Sciences Interdivisional Research Ethics Committee. Upon arrival on the scanning or behavioral site, and after being informed about the study’s aims and procedures, all participants above 16 y of age or the participants’ parents/guardians if the children were below 16 y of age, were asked to complete the relevant consent form. In addition to the consent form completed by the parent/guardian, children below 16 y of age were given an age-appropriate assent form. This form included several statements such as “I have read the study information sheet and I had the opportunity to consider the information, ask questions and have had these answered satisfactorily.” The children were asked to put their initials next to each statement if they agreed and signed the form.

Outliers were defined as cases with a score of three SDs beyond the mean (in the brain or behavioral measures, and in the residuals in the regression model), and were excluded from the analysis. Cases with missing scores were excluded from a given analysis only if the variables involved featured missing scores/excluded cases (*SI Appendix* 6).

The behavioral test at T2 after the neuroimaging data acquisition and behavioral testing was run on 39 participants. This attrition rate was due to the inability of the participants to return for testing as most of them had graduated and moved away from the testing location to pursue a university degree.

### Math Group.

The math group, the single dependent variable of this work, was coded as a dichotomous variable (1: math group vs. 0: nonmath group). In the A-level cohort, the math group consisted of students engaged in A-level math (*n* = 49), while the nonmath group consisted of students who were not engaged in A-level math (*n* = 38). In the pre-A-level cohort, the math group was composed of students who indicated their willingness to study math as one of their A-level subjects (*n* = 21) or to avoid such a subject (*n* = 21). The participants confirmed at a later date that they had taken such a decision.

### MRI Data Acquisition and Preprocessing.

All MRI data were acquired at the Oxford Centre for Functional MRI of the Brain (FMRIB) on a 3T Siemens MAGNETOM Prisma MRI System equipped with a 32-channel receive-only head coil.

### Structural MRI.

Anatomical high-resolution T1-weighted scans were acquired using an MPRAGE sequence consisting of 192 slices (repetition time [TR] = 1,900 ms; echo time [TE] = 3.97 ms; voxel size = 1 × 1 × 1 mm).

### Magnetic Resonance Spectroscopy.

Spectra were measured by semiadiabatic localization using an adiabatic selective refocusing (semi-LASER) sequence (TE = 32 ms; TR = 3.5 s; 32 averages) (87, 88) and variable power radio frequency (RF) pulses with optimized relaxation delays (VAPOR), water suppression, and outer volume saturation. Unsuppressed water spectra acquired from the same volume of interest were used to remove residual eddy current effects and to reconstruct the phased array spectra with MRspa (https://www.cmrr.umn.edu/downloads/mrspa/). Two 20 × 20 × 20 mm^3^ voxels of interest were manually centered in the left IPS and the MFG based on the individual’s T1-weighted image while the participant was lying down in the MR scanner. Acquisition time per voxel was 10 to 15 min, including sequence planning and shimming.

MRS neurotransmitters were quantified with an LCModel (89) using a basis set of simulated spectra generated based on previously reported chemical shifts and coupling constants based on a VeSPA (versatile simulation, pulses, and analysis) simulation library (90). Simulations were performed using the same RF pulses and sequence timings as in the aforementioned 3T system. Eight LCModel-simulated macromolecule resonances were included in the analysis at the following positions: 0.91, 1.21, 1.43, 1.67, 1.95, 2.08, 2.25, and 3 ppm (91). Absolute neurochemical concentrations were extracted from the spectra using a water signal as an internal concentration reference.

The exclusion criteria for the data were 1) Cramér–Rao bounds and 2) the signal-to-noise ratio (SNR) (92). Neurochemicals quantified with Cramér–Rao lower bounds (CRLB, the estimated error of the neurochemical quantification) >50% were classified as undetected. Additionally, we excluded cases with an SNR ranging beyond 3 SDs (per region, per neurochemical, per age group), and cases with a neurochemical score beyond 3 SDs, as mentioned previously.

Absolute neurochemical concentrations were then scaled based on the structural properties of the selected regions and on the predefined values shown in Eq. **1** (89); these predefined scaling values were therefore determined prior to data collection. To quantify the structural properties, we segmented the images into different tissue classes including gray matter (GM), white matter (WM), and cerebrospinal fluid (CSF) using the SPM12 segmentation facility. Next, we calculated the number of GM, WM, and CSF voxels within the two masks of interest separately around the left IPS and MFG in their native space. Subsequently, we divided these six numbers (GM, WM, and CSF for IPS and MFG) by the total number of GM, WM, and CSF voxels to obtain the corresponding GM, WM, and CSF fraction values per participant and region. As a final computation step, we scaled the absolute neurotransmitter values to these structural fractions using the following LCModel (89) computation:

Tissue  corrected concentration=[(43,300/55,556×GM fraction+35,880/55,556×WM fraction+1×CSF fraction)/(1−CSF fraction)]×absolute neurochemical concentration. [1]

We additionally calculated the relative neurotransmitter concentration, which was calculated as the absolute neurotransmitter concentration divided by the concentration of total creatine, where tCr = creatine and phosphocreatine concentration. The neurotransmitter concentrations were referenced to total creatine since 1) creatine is commonly used as a reference and widely accepted as an internal reference standard; and 2) its signal shares the same imperfections (e.g., frequency drift, phase drift, and subject motion) as the signal of GABA and glutamate, as all concentrations are acquired simultaneously (47). We obtained similar results when we scaled GABA and glutamate to creatine and phosphocreatine (tCr, a relative concentration measure).

### Resting fMRI.

Functional images were acquired with a multiband acquisition sequence (multiband acceleration factor = 6; TR = 933 ms; TE = 33.40 ms; flip angle 64°; number of slices = 72; voxel dimension = 2 × 2 × 2; number of volumes = 380). Resting fMRI data were preprocessed and analyzed using the CONN toolbox (http://www.nitrc.org/projects/conn, RRID:SCR_009550) (93) in SPM12 (Wellcome Department of Imaging Neuroscience, Institute of Neurology, London, UK) and the default MNI-space direct normalization preprocessing pipeline. Functional volumes were motion corrected, slice-time corrected, segmented, normalized to a standardized (MNI) template, and spatially smoothed with a Gaussian kernel (8 mm full width at half maximum [FWHM]) and bandpass filter (0.01 Hz to infinity). Moreover, our preprocessing also involved outlier identification (>2 mm) and denoising where the default options were used. We excluded cases where 1) the outlier-identification step excluded more than 5% of the scans, and/or 2) the voxel-to-voxel correlation histogram was significantly nonzero (*r* > 0.15). To examine the effect of the math group, or the effect of neurochemical concentrations, on the brain network level, we employed seed-to-voxel analyses. Due to the findings (see *Results*), our seed was the left MFG. For testing significance, we utilized an initial voxel-wise uncorrected threshold *P* < 0.001 and a cluster-level FDR corrected to *P* < 0.05, which are also the default values of the CONN toolbox.

### Behavioral Tests.

We additionally assessed math ability and anxiety because these are common determinants of the decision to study A-level math (7, 37). The inclusion of these behavioral measures allowed us to examine whether our neural markers could explain the A-level math decision and advanced math learning over and above these common determinants (7, 8).

### Math Ability Assessment.

Math ability was assessed from the mathematical reasoning and the numerical operations tests in a standardized battery (the Wechsler Individual Achievement Test-II UK) (94). The mathematical reasoning test is composed of math problems requiring participants to create a mental model of the problem, extract relevant information, and then select and execute the appropriate operation (95). However, the numerical operation test is composed of written arithmetic problems, which require the implementation of arithmetic procedures. We calculated the proportion of correct responses in each math test for each participant. Note that the same results are obtained for the raw scores, as the proportion of correct responses is based on dividing the raw scores by the number of an overall fixed number of items in each test.

### Math Anxiety Assessment.

Math anxiety was assessed with the single-item math anxiety (SIMA) scale (96), in which higher scores indicate higher levels of math-related anxiety.

### General Cognitive Ability.

We measured general cognitive abilities using the raw scores matrix reasoning subtest of the Wechsler Abbreviated Intelligence Scale (97).

### Statistical Analyses.

To calculate the differences in math ability and math anxiety between the math and nonmath groups, we performed an independent sample *t* test. For effect size, we used Cohen’s d based on the formula (Mean_1_ − Mean_2_)/sqrt((SD_1_^2 + SD_2_^2)/2). To classify the math vs. nonmath groups, we also executed a binary logistic regression. For an effect size in this statistical analysis, we used standardized regression (β) coefficients. We also report the corresponding exponentiation of the partially standardized (pβ) coefficient, which is an odds ratio and is easier to interpret than the standardized regression (β) coefficient, which is in log-odds units. We also examined the equality of variances between the math and nonmath groups using the Levene's test for equality of variances; the variance did not differ between the math and nonmath groups in A levels [MFG GABA *F*(81) = 0.005, *P* = 0.94; MFG glutamate *F*(83) = 0.0001, *P* = 0.99; IPS GABA *F*(83) = 0.298, *P* = 0.59; IPS glutamate *F*(82) = 1.051, *P* = 0.31].

## Supplementary Material

Supplementary File

## Data Availability

Anonymized RAW imaging files, behavioral scores data have been deposited in XNAT Central (https://central.xnat.org/data/projects/PN21).

## References

[1] J. Beddington ., The mental wealth of nations. Nature 455, 1057–1060 (2008). 18948946 10.1038/4551057a

[2] K. Gerardi, L. Goette, S. Meier, Numerical ability predicts mortgage default. Proc. Natl. Acad. Sci. U.S.A. 110, 11267–11271 (2013). 23798401 10.1073/pnas.1220568110 PMC3710828

[3] G. J. Duncan ., School readiness and later achievement. Dev. Psychol. 43, 1428–1446 (2007). 18020822 10.1037/0012-1649.43.6.1428

[4] S. J. Ritchie, T. C. Bates, Enduring links from childhood mathematics and reading achievement to adult socioeconomic status. Psychol. Sci. 24, 1301–1308 (2013). 23640065 10.1177/0956797612466268

[5] S. Parsons, J. Bynner, Does Numeracy Matter More (NRDC, London, 2005).

[6] M. Adkins, A. Noyes, Reassessing the economic value of advanced level mathematics. Br. Educ. Res. J. 42, 93–116 (2016).

[7] R. W. Lent, F. G. Lopez, K. J. Bieschke, Predicting mathematics-related choice and success behaviors: Test of an expanded social cognitive model. J. Vocat. Behav. 42, 223–236 (1993).

[8] G. Hackett, Role of mathematics self-efficacy in the choice of math-related majors of college women and men: A path analysis. J. Couns. Psychol. 32, 47–56 (1985).

[9] M. Arsalidou, M. Pawliw-Levac, M. Sadeghi, J. Pascual-Leone, Brain areas associated with numbers and calculations in children: Meta-analyses of fMRI studies. Dev. Cogn. Neurosci. 30, 239–250 (2018). 28844728 10.1016/j.dcn.2017.08.002 PMC6969084

[10] M. Arsalidou, M. J. Taylor, Is 2+2=4? Meta-analyses of brain areas needed for numbers and calculations. Neuroimage 54, 2382–2393 (2011). 20946958 10.1016/j.neuroimage.2010.10.009

[11] K. Moeller, K. Willmes, E. Klein, A review on functional and structural brain connectivity in numerical cognition. Front. Hum. Neurosci. 9, 227 (2015). 26029075 10.3389/fnhum.2015.00227 PMC4429582

[12] M. Rosenberg-Lee, M. Barth, V. Menon, What difference does a year of schooling make? Maturation of brain response and connectivity between 2nd and 3rd grades during arithmetic problem solving. Neuroimage 57, 796–808 (2011). 21620984 10.1016/j.neuroimage.2011.05.013 PMC3165021

[13] V. Menon, “Memory and cognitive control circuits in mathematical cognition and learning” in Progress in Brain Research, M. Cappelletti, W. Fias, Eds. (Elsevier, 2016), 227, pp. 159–186. 10.1016/bs.pbr.2016.04.026 PMC5811224 27339012

[14] B. R. Sajja, J. S. Wolinsky, P. A. Narayana, Proton magnetic resonance spectroscopy in multiple sclerosis. Neuroimaging Clin. N. Am. 19, 45–58 (2009). 19064199 10.1016/j.nic.2008.08.002 PMC2615006

[15] B. Butterworth, Y. Kovas, Understanding neurocognitive developmental disorders can improve education for all. Science 340, 300–305 (2013). 23599478 10.1126/science.1231022

[16] L. Kaufmann, G. Wood, O. Rubinsten, A. Henik, Meta-analyses of developmental fMRI studies investigating typical and atypical trajectories of number processing and calculation. Dev. Neuropsychol. 36, 763–787 (2011). 21761997 10.1080/87565641.2010.549884

[17] K. L. Mills, C. K. Tamnes, Methods and considerations for longitudinal structural brain imaging analysis across development. Dev. Cogn. Neurosci. 9, 172–190 (2014). 24879112 10.1016/j.dcn.2014.04.004 PMC6989768

[18] S. Qin ., Hippocampal-neocortical functional reorganization underlies children’s cognitive development. Nat. Neurosci. 17, 1263–1269 (2014). 25129076 10.1038/nn.3788 PMC4286364

[19] A. Nieder, A Brain for Numbers: The Biology of the Number Instinct (MIT Press, 2019).

[20] J. F. Werker, T. K. Hensch, Critical periods in speech perception: New directions. Annu. Rev. Psychol. 66, 173–196 (2015). 25251488 10.1146/annurev-psych-010814-015104

[21] N. Gogolla, A. E. Takesian, G. Feng, M. Fagiolini, T. K. Hensch, Sensory integration in mouse insular cortex reflects GABA circuit maturation. Neuron 83, 894–905 (2014). 25088363 10.1016/j.neuron.2014.06.033 PMC4177076

[22] E. Cherubini, J. L. Gaiarsa, Y. Ben-Ari, GABA: An excitatory transmitter in early postnatal life. Trends Neurosci. 14, 515–519 (1991). 1726341 10.1016/0166-2236(91)90003-d

[23] M. M. Silveri ., Frontal lobe γ-aminobutyric acid levels during adolescence: Associations with impulsivity and response inhibition. Biol. Psychiatry 74, 296–304 (2013). 23498139 10.1016/j.biopsych.2013.01.033 PMC3695052

[24] D. A. McCormick, GABA as an inhibitory neurotransmitter in human cerebral cortex. J. Neurophysiol. 62, 1018–1027 (1989). 2573696 10.1152/jn.1989.62.5.1018

[25] L. D. Selemon, A role for synaptic plasticity in the adolescent development of executive function. Transl. Psychiatry 3, e238 (2013). 23462989 10.1038/tp.2013.7 PMC3625918

[26] M. Amalric, S. Dehaene, Origins of the brain networks for advanced mathematics in expert mathematicians. Proc. Natl. Acad. Sci. U.S.A. 113, 4909–4917 (2016). 27071124 10.1073/pnas.1603205113 PMC4983814

[27] V. Menon, Arithmetic in the Child and Adult Brain (Oxford University Press, 2015).

[28] M. Delazer ., Learning complex arithmetic—An fMRI study. Brain Res. Cogn. Brain Res. 18, 76–88 (2003). 14659499 10.1016/j.cogbrainres.2003.09.005

[29] S. M. Rivera, A. L. Reiss, M. A. Eckert, V. Menon, Developmental changes in mental arithmetic: Evidence for increased functional specialization in the left inferior parietal cortex. Cereb. Cortex 15, 1779–1790 (2005). 15716474 10.1093/cercor/bhi055

[30] A. Snowball ., Long-term enhancement of brain function and cognition using cognitive training and brain stimulation. Curr. Biol. 23, 987–992 (2013). 23684971 10.1016/j.cub.2013.04.045 PMC3675670

[31] L. Zamarian, A. Ischebeck, M. Delazer, Neuroscience of learning arithmetic–Evidence from brain imaging studies. Neurosci. Biobehav. Rev. 33, 909–925 (2009). 19428500 10.1016/j.neubiorev.2009.03.005

[32] K. Supekar ., Neural predictors of individual differences in response to math tutoring in primary-grade school children. Proc. Natl. Acad. Sci. U.S.A. 110, 8230–8235 (2013). 23630286 10.1073/pnas.1222154110 PMC3657798

[33] T. Iuculano ., Cognitive tutoring induces widespread neuroplasticity and remediates brain function in children with mathematical learning disabilities. Nat. Commun. 6, 8453 (2015). 26419418 10.1038/ncomms9453 PMC4598717

[34] K. Kucian ., Mental number line training in children with developmental dyscalculia. Neuroimage 57, 782–795 (2011). 21295145 10.1016/j.neuroimage.2011.01.070

[35] M. Cappelletti ., Transfer of cognitive training across magnitude dimensions achieved with concurrent brain stimulation of the parietal lobe. J. Neurosci. 33, 14899–14907 (2013). 24027289 10.1523/JNEUROSCI.1692-13.2013 PMC3771029

[36] R. Cohen Kadosh, S. Soskic, T. Iuculano, R. Kanai, V. Walsh, Modulating neuronal activity produces specific and long-lasting changes in numerical competence. Curr. Biol. 20, 2016–2020 (2010). 21055945 10.1016/j.cub.2010.10.007 PMC2990865

[37] R. Hembree, The nature, effects, and relief of mathematics anxiety. J. Res. Math. Educat. 21, 33–46 (1990).

[38] D. Ansari, Effects of development and enculturation on number representation in the brain. Nat. Rev. Neurosci. 9, 278–291 (2008). 18334999 10.1038/nrn2334

[39] V. Menon, S. M. Rivera, C. D. White, G. H. Glover, A. L. Reiss, Dissociating prefrontal and parietal cortex activation during arithmetic processing. Neuroimage 12, 357–365 (2000). 10988030 10.1006/nimg.2000.0613

[40] R. Cohen Kadosh ., Virtual dyscalculia induced by parietal-lobe TMS impairs automatic magnitude processing. Curr. Biol. 17, 689–693 (2007). 17379521 10.1016/j.cub.2007.02.056

[41] J. M. Chein, W. Schneider, The brain’s learning and control architecture. Curr. Dir. Psychol. Sci. 21, 78–84 (2012).

[42] K. Aydin ., Increased gray matter density in the parietal cortex of mathematicians: A voxel-based morphometry study. AJNR Am. J. Neuroradiol. 28, 1859–1864 (2007). 17921236 10.3174/ajnr.A0696 PMC8134244

[43] T. Popescu ., The brain-structural correlates of mathematical expertise. Cortex 114, 140–150 (2019). 30424836 10.1016/j.cortex.2018.10.009 PMC6996130

[44] R. H. Grabner, B. Rütsche, C. C. Ruff, T. U. Hauser, Transcranial direct current stimulation of the posterior parietal cortex modulates arithmetic learning. Eur. J. Neurosci. 42, 1667–1674 (2015). 25970697 10.1111/ejn.12947

[45] K. Cohen Kadosh, R. Cohen Kadosh, F. Dick, M. H. Johnson, Developmental changes in effective connectivity in the emerging core face network. Cereb. Cortex 21, 1389–1394 (2011). 21045001 10.1093/cercor/bhq215 PMC3094719

[46] K. Cohen Kadosh, M. H. Johnson, F. Dick, R. Cohen Kadosh, S.-J. Blakemore, Effects of age, task performance, and structural brain development on face processing. Cereb. Cortex 23, 1630–1642 (2013). 22661406 10.1093/cercor/bhs150 PMC3446867

[47] K. Cohen Kadosh, B. Krause, A. J. King, J. Near, R. Cohen Kadosh, Linking GABA and glutamate levels to cognitive skill acquisition during development. Hum. Brain Mapp. 36, 4334–4345 (2015). 26350618 10.1002/hbm.22921 PMC4832309

[48] E. C. Porges ., Frontal gamma-aminobutyric acid concentrations are associated with cognitive performance in older adults. Biol. Psychiatry Cogn. Neurosci. Neuroimaging 2, 38–44 (2017). 28217759 10.1016/j.bpsc.2016.06.004 PMC5312683

[49] C. Lüscher, R. C. Malenka, NMDA receptor-dependent long-term potentiation and long-term depression (LTP/LTD). Cold Spring Harb. Perspect. Biol. 4, a005710 (2012). 22510460 10.1101/cshperspect.a005710 PMC3367554

[50] F. S. Nugent, E. C. Penick, J. A. Kauer, Opioids block long-term potentiation of inhibitory synapses. Nature 446, 1086–1090 (2007). 17460674 10.1038/nature05726

[51] P. E. Castillo, C. Q. Chiu, R. C. Carroll, Long-term plasticity at inhibitory synapses. Curr. Opin. Neurobiol. 21, 328–338 (2011). 21334194 10.1016/j.conb.2011.01.006 PMC3092861

[52] D. T. Stuss, M. P. Alexander, Executive functions and the frontal lobes: A conceptual view. Psychol. Res. 63, 289–298 (2000). 11004882 10.1007/s004269900007

[53] M. B. Jurado, M. Rosselli, The elusive nature of executive functions: A review of our current understanding. Neuropsychol. Rev. 17, 213–233 (2007). 17786559 10.1007/s11065-007-9040-z

[54] R. C. Chan, D. Shum, T. Toulopoulou, E. Y. Chen, Assessment of executive functions: Review of instruments and identification of critical issues. Arch. Clin. Neuropsychol. 23, 201–216 (2008). 18096360 10.1016/j.acn.2007.08.010

[55] J. A. Alvarez, E. Emory, Executive function and the frontal lobes: A meta-analytic review. Neuropsychol. Rev. 16, 17–42 (2006). 16794878 10.1007/s11065-006-9002-x

[56] B. Krause, C. Y. Looi, M. Dresler, R. C. Kadosh, The neurochemistry of mathematical genius: Reduced frontal excitation/inhibition balance in an expert calculator. Neuroscience 392, 252–257 (2018). 30114457 10.1016/j.neuroscience.2018.08.002

[57] A. Hampshire, R. Thompson, J. Duncan, A. M. Owen, Lateral prefrontal cortex subregions make dissociable contributions during fluid reasoning. Cereb. Cortex 21, 1–10 (2011). 20483908 10.1093/cercor/bhq085 PMC3000572

[58] J. Duncan, An adaptive coding model of neural function in prefrontal cortex. Nat. Rev. Neurosci. 2, 820–829 (2001). 11715058 10.1038/35097575

[59] A. Diamond, Executive functions. Annu. Rev. Psychol. 64, 135–168 (2013). 23020641 10.1146/annurev-psych-113011-143750 PMC4084861

[60] A. Dowker, Individual Differences in Arithmetic: Implications for Psychology, Neuroscience and Education (Routledge, 2019).

[61] K. Garnett, J. E. Fleischner, Automatization and basic fact performance of normal and learning disabled children. Learn. Disabil. Q. 6, 223–230 (1983).

[62] H. Hu, J. Gan, P. Jonas, Interneurons. Fast-spiking, parvalbumin^+^ GABAergic interneurons: From cellular design to microcircuit function. Science 345, 1255263 (2014). 25082707 10.1126/science.1255263

[63] P. Frangou, M. Correia, Z. Kourtzi, GABA, not BOLD, reveals dissociable learning-dependent plasticity mechanisms in the human brain. eLife 7, e35854 (2018). 30355444 10.7554/eLife.35854 PMC6202049

[64] K. Shibata ., Overlearning hyperstabilizes a skill by rapidly making neurochemical processing inhibitory-dominant. Nat. Neurosci. 20, 470–475 (2017). 28135242 10.1038/nn.4490 PMC5323354

[65] T. Toyoizumi ., A theory of the transition to critical period plasticity: Inhibition selectively suppresses spontaneous activity. Neuron 80, 51–63 (2013). 24094102 10.1016/j.neuron.2013.07.022 PMC3800182

[66] F. Condé, J. S. Lund, D. M. Jacobowitz, K. G. Baimbridge, D. A. Lewis, Local circuit neurons immunoreactive for calretinin, calbindin D-28k or parvalbumin in monkey prefrontal cortex: Distribution and morphology. J. Comp. Neurol. 341, 95–116 (1994). 8006226 10.1002/cne.903410109

[67] P. L. Gabbott, S. J. Bacon, Local circuit neurons in the medial prefrontal cortex (areas 24a,b,c, 25 and 32) in the monkey: II. Quantitative areal and laminar distributions. J. Comp. Neurol. 364, 609–636 (1996). 8821450 10.1002/(SICI)1096-9861(19960122)364:4<609::AID-CNE2>3.0.CO;2-7

[68] L. M. Schultz, GABAergic inhibitory processes and hippocampal long-term potentiation. Neuroscientist 3, 226–236 (1997).

[69] W. Inoue ., Noradrenaline is a stress-associated metaplastic signal at GABA synapses. Nat. Neurosci. 16, 605–612 (2013). 23563580 10.1038/nn.3373 PMC3984240

[70] J. Arrubla, D. H. Tse, C. Amkreutz, I. Neuner, N. J. Shah, GABA concentration in posterior cingulate cortex predicts putamen response during resting state fMRI. PLoS One 9, e106609 (2014). 25184505 10.1371/journal.pone.0106609 PMC4153676

[71] D. Kapogiannis, D. A. Reiter, A. A. Willette, M. P. Mattson, Posteromedial cortex glutamate and GABA predict intrinsic functional connectivity of the default mode network. Neuroimage 64, 112–119 (2013). 23000786 10.1016/j.neuroimage.2012.09.029 PMC3801193

[72] C. J. Stagg ., Local GABA concentration is related to network-level resting functional connectivity. eLife 3, e01465 (2014). 24668166 10.7554/eLife.01465 PMC3964822

[73] P. Bonifazi ., GABAergic hub neurons orchestrate synchrony in developing hippocampal networks. Science 326, 1419–1424 (2009). 19965761 10.1126/science.1175509

[74] U. Rutishauser, J.-J. Slotine, R. J. Douglas, Competition through selective inhibitory synchrony. Neural Comput. 24, 2033–2052 (2012). 22509969 10.1162/NECO_a_00304

[75] T. Reed, R. Cohen Kadosh, Transcranial electrical stimulation (tES) mechanisms and its effects on cortical excitability and connectivity. J. Inherit. Metab. Dis. 41, 1123–1130 (2018). 10.1007/s10545-018-0181-4 PMC6326965 30006770

[76] H. C. Barron ., Unmasking latent inhibitory connections in human cortex to reveal dormant cortical memories. Neuron 90, 191–203 (2016). 26996082 10.1016/j.neuron.2016.02.031 PMC4826438

[77] A. Hone-Blanchet, R. A. Edden, S. Fecteau, Online effects of transcranial direct current stimulation in real time on human prefrontal and striatal metabolites. Biol. Psychiatry 80, 432–438 (2016). 26774968 10.1016/j.biopsych.2015.11.008 PMC5512102

[78] K. Kihara, H. M. Kondo, J. I. Kawahara, Differential contributions of GABA concentration in frontal and parietal regions to individual differences in attentional blink. J. Neurosci. 36, 8895–8901 (2016). 27559171 10.1523/JNEUROSCI.0764-16.2016 PMC6601901

[79] S. Kim, M. C. Stephenson, P. G. Morris, S. R. Jackson, tDCS-induced alterations in GABA concentration within primary motor cortex predict motor learning and motor memory: a 7 T magnetic resonance spectroscopy study. Neuroimage 99, 237–243 (2014). 24904994 10.1016/j.neuroimage.2014.05.070 PMC4121086

[80] C. Lunghi, U. E. Emir, M. C. Morrone, H. Bridge, Short-term monocular deprivation alters GABA in the adult human visual cortex. Curr. Biol. 25, 1496–1501 (2015). 26004760 10.1016/j.cub.2015.04.021 PMC5040500

[81] C. J. Stagg ., Polarity-sensitive modulation of cortical neurotransmitters by transcranial stimulation. J. Neurosci. 29, 5202–5206 (2009). 19386916 10.1523/JNEUROSCI.4432-08.2009 PMC6665468

[82] D. B. Terhune ., Phosphene perception relates to visual cortex glutamate levels and covaries with atypical visuospatial awareness. Cereb. Cortex 25, 4341–4350 (2015). 25725043 10.1093/cercor/bhv015 PMC4816785

[83] F. Sella, E. Sader, S. Lolliot, R. Cohen Kadosh, Basic and advanced numerical performances relate to mathematical expertise but are fully mediated by visuospatial skills. J. Exp. Psychol. Learn. Mem. Cogn. 42, 1458–1472 (2016). 26913930 10.1037/xlm0000249 PMC5008436

[84] N. Attridge, M. Inglis, Advanced mathematical study and the development of conditional reasoning skills. PLoS One 8, e69399 (2013). 23869241 10.1371/journal.pone.0069399 PMC3711803

[85] L. Phil ., The LEGO Movie (DVD 2-disc special edition, Burbank, CA: Warner Home Video (2014).

[86] R. C. Oldfield, The assessment and analysis of handedness: The Edinburgh inventory. Neuropsychologia 9, 97–113 (1971). 5146491 10.1016/0028-3932(71)90067-4

[87] D. K. Deelchand ., Two-site reproducibility of cerebellar and brainstem neurochemical profiles with short-echo, single-voxel MRS at 3T. Magn. Reson. Med. 73, 1718–1725 (2015). 24948590 10.1002/mrm.25295 PMC4272339

[88] G. Öz, I. Tkáč, Short-echo, single-shot, full-intensity proton magnetic resonance spectroscopy for neurochemical profiling at 4 T: validation in the cerebellum and brainstem. Magn. Reson. Med. 65, 901–910 (2011). 21413056 10.1002/mrm.22708 PMC3827699

[89] S. W. Provencher, Automatic quantitation of localized in vivo 1H spectra with LCModel. NMR Biomed. 14, 260–264 (2001). 11410943 10.1002/nbm.698

[90] B. Soher, P. Semanchuk, D. Todd, J. Steinberg, K. Young, VeSPA: Integrated applications for RF pulse design, spectral simulation and MRS data analysis. Proc. Intl. Soc. Mag. Reson. Med. 19, 1410 (2011). 10.1002/mrm.29686 PMC10330446 37183778

[91] B. Schaller, L. Xin, C. Cudalbu, R. Gruetter, Quantification of the neurochemical profile using simulated macromolecule resonances at 3 T. NMR Biomed. 26, 593–599 (2013). 23413241 10.1002/nbm.2896

[92] U. E. Emir, P. J. Tuite, G. Öz, Elevated pontine and putamenal GABA levels in mild-moderate Parkinson disease detected by 7 tesla proton MRS. PLoS One 7, e30918 (2012). 22295119 10.1371/journal.pone.0030918 PMC3266292

[93] S. Whitfield-Gabrieli, A. Nieto-Castanon, Conn: A functional connectivity toolbox for correlated and anticorrelated brain networks. Brain Connect. 2, 125–141 (2012). 22642651 10.1089/brain.2012.0073

[94] D. Wechsler, Wechsler Individual Achievement Test—Second UK Edition (The Psychological Corporation, London, 2005).

[95] D. Lucangeli, P. E. Tressoldi, M. Cendron, Cognitive and metacognitive abilities involved in the solution of mathematical word problems: Validation of a comprehensive model. Contemp. Educ. Psychol. 23, 257–275 (1998). 9665790 10.1006/ceps.1997.0962

[96] M. I. Núñez-Peña, G. Guilera, M. Suárez-Pellicioni, The single-item math anxiety scale: An alternative way of measuring mathematical anxiety. J. Psychoed. Assess. 32, 306–317 (2014).

[97] D. Wechsler, WASI-II: Wechsler Abbreviated Scale of Intelligence (PsychCorp, 2011).

