# Supplementary material for: The impact of a lack of mathematical education on brain development and future attainment
Source: Proc Natl Acad Sci U S A. 2021 Jun 7;118(24):e2013155118. doi: 10.1073/pnas.2013155118 (PMC8214709; doi:10.1073/pnas.2013155118)
Supplement: Supplementary File [file pnas.2013155118.sapp.pdf]

SI Appendix

SI Appendix 1. Mean and standard deviations in parentheses for maths ability (mathematical reasoning, numerical operations) and maths anxiety in the A-level maths and A-level non-maths students (top half) and the pre-A-level maths and pre-A-level non-maths students (bottom half).

	**A-level cohort**		
	**Mathematical Reasoning**	**Numerical Operations**	**Maths Anxiety**
Maths	92.72 (4.69)	90.74 (7.35)	3.35 (1.88)
Non-maths	87.41 (5.94)	79.78 (10.92)	4.81 (2.23)

	**Pre A-level cohort**		
	**Mathematical Reasoning**	**Numerical Operations**	**Maths Anxiety**
Maths	92.42 (3.47)	87.04 (6.26)	3.56 (1.92)
Non-maths	83.93 (8.05)	70.37 (12.66)	4.12 (2)

SI Appendix 2

We performed additional analyses to examine whether gender affects the capacity of MFG GABA concentration in classifying adolescents who lack an A-level maths education vs. those who did not. MFG GABA concentration was successful in classifying those who did not study maths even after controlling for gender (N=83, β=-.25, P=.028, Exp(pβ)=.57, Exp(β)=.78).

SI Appendix 3

Additional analyses examining the specificity of the findings in the main text. First, we compared the students who lack a maths education vs. those who did not in the variables presented in the first column of the table below. We discovered that students who lack a maths education were enrolled in fewer A-level subjects (M=3.29) than the students who continued their maths education (M=3.73, Mann-Whitney U=547, P<.001). Moreover, there existed gender differences between the students who lacked a maths education (30 females, 8 males) and the students who did not lack maths (26 females, 23 males), but these gender differences did not compromise the main result, as can be seen in **SI Appendix 3**.

	Df	P	T	Cohen's d
A-level duration	82	0.21	1.26	0.28
Matrix Reasoning	82	0.12	-1.58	-0.35
Age during scanning (in days)	85	0.57	0.56	0.12
	Df	P	χ^2	
Gender	1	0.012	6.253	

To examine whether the contribution of MFG GABA is confounded by total A-level subjects, we ran a binary logistic regression classifying whether a student lacks a maths education based on MFG GABA (N=83, β=-0.23, P=0.044, Exp(pβ)=.59, Exp(β)=.79) and total A-level subjects (N=83, β=-0.34, P=0.003, Exp(pβ)=.46, Exp(β)=.71). As can be seen from the results, MFG GABA was a successful classifier after controlling for total A-level subjects.

Next, we examined the impact of other A-level subjects associated with A-level maths (i.e., biology, chemistry, and physics) on our results by running a binary logistic regression classifying whether a student lacks a maths education based on MFG GABA (N=82, β=-0.04, P=0.032, Exp(pβ)=.54, Exp(β)=.96), the choice of biology A-level (N=82, β=0.04, P=0.345, Exp(pβ)=1.81, Exp(β)=1.04), chemistry A-level (N=82, β=-0.1, P=0.01, Exp(pβ)=.2, Exp(β)=.9) and physics A-level (N=82, β=-1.35, P=1, Exp(pβ)=0, Exp(β)=.26). As can be seen from the results, MFG GABA was a successful classifier even after controlling for the choice of these other maths-related subjects.

Furthermore, we run three similar binary logistic regression analyses each classifying whether a student lacks a physics, biology, or chemistry education based on MFG GABA while controlling for the other three A-level subjects. We classified whether a student lacks a physics education based on the choice of biology A-level (N=82, β=.17, P=0.004, Exp(pβ)=16.95, Exp(β)=1.18), chemistry A-level (N=82, β=-.1, P=.08, Exp(pβ)=.2, Exp(β)=.91), maths A-level (N=82, β=-1.18, P=1, Exp(pβ)=0, Exp(β)=.31), and MFG GABA (N=82, β=-.02, P=0.33, Exp(pβ)=.69, Exp(β)=.98). Similarly, we classified whether a student lacks a biology education based on the choice of a chemistry A-level (N=82, β=-0.97, P<0.001, Exp(pβ)=.04, Exp(β)=.38) physics A-level (N=82, β=0.94, P=.002, Exp(pβ)=22.7, Exp(β)=2.55), maths A-level (N=82, β=0.32, P=0.1, Exp(pβ)=2.9, Exp(β)=1.38), and MFG GABA (N=82, β=-0.09, P=0.32, Exp(pβ)=.74, Exp(β)=.92). Last, we classified whether a student lacks a chemistry education based on the choice of a biology A-level (N=82, β=-0.99, P<0.001, Exp(pβ)=.04, Exp(β)=.37), physics A-level (N=82, β=-0.64, P=.03, Exp(pβ)=.13, Exp(β)=.53), maths A-level (N=82, β=-0.54, P=0.01, Exp(pβ)=.17, Exp(β)=.58), and MFG GABA (N=82, β=-0.001, P=0.99, Exp(pβ)=1, Exp(β)=1). Taken together, these analyses suggest that MFG GABA classifies a maths education specifically.

We compared the Pre-A-level students who decided to not choose maths as part of their A-level vs. those who decided to choose maths in the variables presented in the first column of the table below.

	Df	P	T	Cohen's d
Matrix Reasoning	39	0.0013	-3.4561	-1.0676
Age during scanning (days)	40	0.2638	1.1333	0.3578
	Df	P	χ^2	
Gender	1	0.12	2.471	

To examine whether the contribution of MFG GABA is confounded by matrix reasoning, we ran a binary logistic regression classifying whether a student decided to not enrol in a maths education based on MFG GABA (N=35, β=-0.01, P=0.94, Exp(pβ)=.97, Exp(β)=.99) and matrix reasoning (N=35, β=-0.52, P=0.007, Exp(pβ)=.25, Exp(β)=.59), but only matrix reasoning was significant.

SI Appendix 4

One potential concern is that our null results with MFG GABA in the case of pre-A-level students are due to a smaller sample size compared to the A-level students, which may have resulted in insufficient statistical power to detect such an effect. However, we note that the standardized beta weights in the two experiments are in opposing polarities (A-levels' β=-.3, pre-A-levels' β=.14), which suggests that the lack of effect in the pre-A-levels is not due to insufficient statistical power. Moreover, we conducted an additional analysis for which we included both A-level and pre-A-level students as a factor together with MFG GABA to predict their maths decision. The interaction between group (A-level, pre-A-level) and MFG GABA was significant (N=119, β=-.44, P=.042, Exp(pβ)=.39, Exp(β)=.65), highlighting the predictive power of MFG GABA in the maths decision of A-level students only.

SI Appendix 5

While not of direct interest to our research question in this manuscript, another question is how our results are explained by non-mathematical factors, with performance IQ being a prime candidate (1, 2). To explore this issue, we entered into our original analysis, as described in the main text as aiming to predict mathematical reasoning at T2, the scores from a performance IQ test assessing the participants' general cognitive ability using the matrix reasoning test (3). The value of adding matrix reasoning lies in its link to performance intelligence, which is associated with mathematical abilities (4). While the performance IQ score at T1 did not predict the mathematical reasoning score at T2 (β=-.11, t(31)=-1.16, P=.25), the performance IQ score at T2 did predict the mathematical reasoning score at T2 (β=.24, t(31)=2.48, P=.019). Notably, the MFG GABA still predicted mathematical reasoning at T2 even after entering the performance IQ scores from T1 and T2 into the regression equation (β=.3, t(31)=3.11, P=.004). Of note is that the mean (across participants) MNI coordinates for the MFG region in the A-level cohort was x=-29.6, y=32.8, z=19.6 which is close, although slightly medial, to the peak coordinates of the resulting regions of the extended multiple demand network in a recent meta-analysis (left MFG/IFS: x=−44, y=32, z=22) (5).

SI Appendix 6

The number of participants excluded in each of the variables in the two developmental groups (A-level and pre-A-level).

	A-level	Pre A-level
Numerical Operations	1	0
Mathematical Reasoning	2	0
Maths anxiety	1	0
MFG GABA	4	6
MFG Glutamate	2	4
IPS GABA	2	5
IPS Glutamate	3	5

SI Appendix References

1. Sella F, Sader E, Lolliot S, & Cohen Kadosh R (2016) Basic and advanced numerical performances relate to mathematical expertise but are fully mediated by visuospatial skills. *Journal of Experimental Psychology: Learning, Memory, and Cognition* 42(9):1458.
2. Peng P, Wang T, Wang C, & Lin X (2019) A meta-analysis on the relation between fluid intelligence and reading/mathematics: Effects of tasks, age, and social economics status. *Psychological Bulletin* 145(2):189.
3. Wechsler D (2011) *WASI-II: Wechsler abbreviated scale of intelligence* (PsychCorp).
4. Kyttälä M & Lehto JE (2008) Some factors underlying mathematical performance: The role of visuospatial working memory and non-verbal intelligence. *European Journal of Psychology of Education* 23(1):77.
5. Camilleri JA, et al. (2018) Definition and characterization of an extended multiple-demand network. *NeuroImage* 165: 138-147.
